# Lomerizine attenuates LPS-induced acute lung injury by inhibiting the macrophage activation through reducing Ca^2+^ influx

**DOI:** 10.3389/fphar.2023.1236469

**Published:** 2023-08-24

**Authors:** Yunduan Song, Yusen Gou, Jiameng Gao, Dongxin Chen, Haibo Zhang, Wenjuan Zhao, Feng Qian, Ajing Xu, Yao Shen

**Affiliations:** ^1^ Department of Respiratory and Critical Care Medicine, Shanghai Pudong Hospital, Fudan University Pudong Medical Center, Shanghai, China; ^2^ Department of Clinical Laboratory, Tongren Hospital, Shanghai Jiao Tong University School of Medicine, Shanghai, China; ^3^ Shanghai Frontiers Science Center of Drug Target Identification and Delivery, School of Pharmacy, Shanghai Jiao Tong University, Shanghai, China; ^4^ Department of Clinical Pharmacy, Xinhua Hospital Affiliated to Shanghai Jiao Tong University School of Medicine, Shanghai, China

**Keywords:** lomerizine, acute lung injury, calcium, macrophage, cytokine, inflammation

## Abstract

Acute lung injury (ALI) and acute respiratory distress syndrome (ARDS) are life-threatening lung diseases with high mortality rates, predominantly attributable to acute and severe pulmonary inflammation. Lomerizine (LMZ) is a calcium channel blocker previously used in preventing and treating migraine. Here, we found that LMZ inhibited inflammatory responses and lung pathological injury by reducing pulmonary edema, neutrophil infiltration and pro-inflammatory cytokine production in lipopolysaccharide (LPS)-induced ALI mice. In *vitro* experiments, upon treating with LMZ, the expression of interleukin (IL)-1β, IL-6 and tumor necrosis factor (TNF)-α was attenuated in macrophages. The phosphorylation of p38 MAPK, ERK1/2, JNK, and NF-κB p65 was inhibited after LMZ treatment. Furthermore, LPS-induced Ca^2+^ influx was reduced by treating with LMZ, which correlated with inhibition of pro-inflammatory cytokine production. And L-type Ca^2+^ channel agonist Bay K8644 (BK) could restore cytokine generation. In conclusion, our study demonstrated that LMZ alleviates LPS-induced ALI and is a potential agent for treating ALI/ARDS.

## 1 Introduction

Acute lung injury (ALI) is an inflammatory disease mainly characterized by alveolar capillary injury, diffuse interstitial, alveolar edema, and hypoxic respiratory insufficiency after severe infection, trauma, shock and other pulmonary attack (Butt et al., 2016). Acute respiratory distress syndrome (ARDS), the more serious form of ALI, is a life-threatening lung disease with high mortality (Rubenfeld et al., 2005; Matthay et al., 2019). Patients suffering from ALI/ARDS have increased sharply since the global outbreak of novel coronavirus pneumonia (COVID-19) in 2019 (Gao et al., 2021; Meyer et al., 2021; Pfortmueller et al., 2021). Mechanical ventilation remains the only supportive therapy for ALI. However, it does not enhance the quality of life for ALI patients and still carries side effects, including lung infection and lung injury (Gattinoni et al., 2016; Gattinoni et al., 2017; Fan et al., 2018). Nowadays, no effective drugs have been reported for treating acute lung injury (Bernard et al., 1987; Steinberg et al., 2006). Therefore, the development of new drugs for ALI therapy is urgent.

Uncontrollable inflammation is the main factor in acute lung injury, contributed from endothelial, epithelial, and alveolar structure injury, and inflammatory cells infiltration. Macrophages play a crucial role in regulating inflammatory responses in ALI/ARDS (Wynn et al., 2013; Watanabe et al., 2019). Macrophages can be activated to phenotype of M1 or M2 in response to environmental signals from the microenvironment (Saqib et al., 2018). M1 macrophages play a pro-inflammatory role by releasing variety of pro-inflammatory cytokines including interleukin (IL)-1β, IL-6 and tumor necrosis factor (TNF)-α (Lee et al., 2021). While M2 macrophages synthesize and release anti-inflammatory cytokines including transforming growth factor (TGF)-β and IL-10, contributing to anti-inflammatory response and tissue remodeling. The transformation of macrophage function is closely related to the initiation and development of pneumonia (Guan et al., 2023). During the pathology of ALI, toll-like receptors (TLRs) are activated by pathogen-associated molecular patterns in macrophages (Arora et al., 2019). The macrophages immediately shift toward M1 phenotype and release various pro-inflammatory cytokines. And then, these pro-inflammatory cytokines recruit neutrophils into the lung and alveolar lumens (Kolaczkowska and Kubes, 2013). Therefore, macrophage polarization is a practical and promising target for ALI treatment.

Lomerizine (LMZ), also known as KB-2796, is a calcium channel blocker, whose chemical name is 1-[bis(4-fluorophenyl)methyl]-4-(2,3,4-trimethoxybenzyl) piperazine. It was first listed in Japan in 1999, and the product was named Migsis and Teranas (Gaudilliere and Berna, 2000). LMZ possesses better selectivity on cerebrovascular, with a strong protective effect on brain tissue with fewer side effects on the heart and central nervous system than earlier migraine drugs. It is normally used to treat migraine in clinical practice through blocking T-type and L-type calcium channels. Drugs that block calcium channels have been verified to reduce inflammation in previous studies (Shima et al., 2008; Huang et al., 2014). Das et al. found that L-type Ca^2+^ channel (LTCC) blockers amlodipine and verapamil exert anti-inflammatory effects by inhibiting the expression of fibrinogen in macrophages, which inhibits macrophage recruitment (Das et al., 2009). Another LTCC blocker, nifedipine, has been shown to inhibit fibroblast activation by antagonizing the activity of mineralocorticoid receptors (Matsui et al., 2010). However, the therapeutic effect of LMZ in ALI has not been reported.

In this study, we demonstrated that LMZ attenuates LPS-induced ALI by decreasing pro-inflammatory cytokines expression through blocking calcium influx, which regulated by mitogen-activated protein kinase (MAPK) and nuclear factor kappa B (NF-κB) signaling pathways. The results revealed that LMZ has potential as a therapeutic agent for ALI/ARDS.

## 2 Materials and methods

### 2.1 Compound and reagents

Lomerizine was purchased from Absin Bioscience Inc. (Shanghai, China). Lipopolysaccharide (LPS) was purchased from Sigma-Aldrich (St. Louis, United States). Dexamethasone was purchased from Thermo Fisher Scientific (Waltham, MA, United States). Hematoxylin and eosin (H&E) staining kit was purchased from Abcam (Waltham, Boston, United States). Dulbecco’s Modified Eagle Medium (DMEM) and macrophage colony-stimulating factor (M-CSF) was purchased from Thermo Fisher Scientific (Waltham, MA, United States). Penicillin-streptomycin solution (Pen Strep) was purchased from Yeasen Biotechnology Co., Ltd. (Shanghai, China). Fetal Bovine Serum (FBS) was purchased from Cegrogen Biotech (Wupperweg, Germany).

Primers were synthesized by Hua Gene Biotech Co., Ltd. (Shanghai, China). The primary antibody against IκB-α was purchased from Santa Cruz Biotechnology (Santa Cruz, CA, United States). Primary antibodies against iNOS, phospho-NF-κB p65 (Ser536) Rabbit mAb, NF-kappaB p65 Rabbit mAb, phospho-p38 MAPK (Thr180/Tyr182) XP Rabbit mAb, p38 MAPK XP Rabbit mAb, phospho-SAPK/JNK (Thr183/Tyr185) Rabbit mAb, SAPK/JNK Antibody, Phospho-p44/42 MAPK (ERK1/2) (Thr202/Tyr204) Antibody, p44/42 MAPK (ERK1/2) Rabbit mAb, and β-Actin Rabbit Antibody were purchased from Cell Signaling Technology (Danvers, MA, United States).

### 2.2 Animals and treatment

C57BL/6 mice were purchased from JST Laboratory Animal Co., Ltd. (Shanghai, China). The mice were bred under sterile and temperature-controlled conditions for 12 h of light and dark cycle. Mice had free access to water and feed in the experimental period. Mice were housed under specific pathogen-free conditions at the Laboratory Animal Center of Shanghai Jiao Tong University. All procedures involving mice were approved by the Institutional Animal Care and Use Committee of Shanghai Jiao Tong University (A2018075).

Thirty male mice aged 8–10 weeks were selected to establish acute lung injury models. The mice were randomly divided into six groups: PBS group, LPS treatment group, dexamethasone (DEX) treatment group, low dose lomerizine (LMZ) group (10 mg/kg), medium dose LMZ group (20 mg/kg), and high dose LMZ group (40 mg/kg). In PBS group, the neck skin of mice was dissected and sutured. The other groups were given 5 mg/kg LPS via intratracheal administration. Moreover, DEX or various doses of LMZ were injected intraperitoneally 1 h after LPS was administered.

### 2.3 Bronchoalveolar lavage fluid collection and analysis

After 6 h of LPS stimulation, mice were anesthetized, and the trachea was exposed with scissors, then a small cut was gently made in the trachea and a needle was inserted, followed by the injection of 1 mL of cooled PBS, waited for 10 s and then the bronchoalveolar lavage fluid (BALF) was slowly withdrawn. And collected BALF was put on ice and then centrifuged at 4°C for 500 rcf for 5 min (Wu et al., 2021). The total number of cells was counted with a hemocytometer. And the supernatant was analyzed for total protein using a EpiZyme BCA kit (Shanghai, China).

### 2.4 Flow cytometry assay

The suppernatant after centrifugation of BALF was added 300 μL red blood cell lysate (Sangon biotech, China) and mixed well, and rested on ice for 5 min, 3 mL PBS was added, followed by 500 rcf centrifugation at 4°C for 5 min. The supernatant was discarded and 100 μL of PBS containing 2% FBS was added to the supernatant for 30 min. The detectable antibodies were then added and incubated for 30 min. At the end of the incubation, the supernatant was mixed with 1 mL of PBS and centrifuged at 500 rcf for 5 min, then the supernatant was discarded and resuspended in 150 μL of PBS. For neutrophils staining, anti-Ly-6G (clone 1A8, Cat. 551460) was used. For alveolar macrophage staining, PerCP/Cy5.5-F4/80, PE-CD11c, and FITC-MHC class II (I-A/I-E) antibodies were used. All flow cytometric antibodies were obtained from BD Bioscience (San Jose, CA, United States).

### 2.5 Enzyme-linked immunosorbent assay

Supernatants from BALF centrifugation and cell culture supernatants from BMDMs were collected and subsequently assayed for cytokine expression using ELISA kits (R&D System, Minneapolis, MN, United States) according to the manufacturer’s instructions.

### 2.6 Histopathology

The lung tissue was removed from the mouse and soaked in deionized water containing 4% paraformaldehyde for 24 h. The lung tissue was then wrapped in paraffin and cut into 10 μm slices with a slicer after the paraffin had solidified. The slices were stained with H&E staining kits, and images were pictured with an Olympus microscope (BX53, Tokyo, Japan).

### 2.7 Cell culture of BMDMs

Euthanized mice were immersed in 75% ethanol for 2 min, then the skin was cut along the abdominal cavity to expose the lower limbs, and the femur and tibia were removed and transferred to DMEM containing 1% Pen Strep. The bones were held with forceps and then the ends were cut with scissors, and 3 mL of cold PBS was injected into the bones with a syringe, and the cells were collected below in a 50 mL centrifuge tube. The collected cells were centrifuged at 500 rcf for 5 min, the supernatant was discarded and subsequently resuspended in complete medium. Cells were cultured in DMEM supplemented with 10% Fetal Bovine Serum (FBS), 1% Penicillin-streptomycin solution (Pen Strep), and 10 ng/mL M-CSF. After 5 days of culturing, cells were planted. BMDMs were pre-treated with LMZ for 30 min and then challenged with 100 ng/mL LPS for 30 min.

### 2.8 Real-time polymerase chain reaction

Trizol purchased from Invitrogen (CA, United States) to extract the total RNA from cell and tissue samples, and the Nano-Drop 2000 micro-spectrophotometer was used for RNA quantification. The cDNA was prepared by the reverse transcription according to the instructions of the Toyobo reverse transcription kit (Toyobo, Osaka, Japan) and amplified by SYBR Green RT-PCR Master Mix kit (Toyobo, Osaka, Japan) on the StepOne Plus system (Thermo Fisher Scientific, Waltham, MA, United States). Glyceraldehyde-3-Phosphate Dehydrogenase (*Gapdh*) to test the gene expression data and normalize mRNA levels of different inflammatory factors. The primer sequences used in the reaction are listed in Table 1.

**TABLE 1:** Specific primer sequences used for RT-PCR.

Gene	Forward primer	Reverse primer
*Gapdh*	5′-CAT​CAC​TGC​CAC​CCA​GAA​GAC​TG-3′	5′-ATG​CCA​GTG​AGC​TTC​CCG​TTC​AG-3′
*Tnfα*	5′-TGG​ACC​TTC​CAG​GAT​GAG​GAC​A-3′	5′-GCC​ATA​GAA​CTG​ATG​AGA​GGG​AG -3′
*Il1β*	5′-TGG​ACC​TTC​CAG​GAT​GAG​GAC​A-3′	5′-GTT​CAT​CTC​GGA​GCC​TGT​AGT​G-3′
*Il6*	5′-TAC​CAC​TTC​ACA​AGT​CGG​AGG​C-3′	5′-CTG​CAA​GTG​CAT​CAT​CGT​TGT​TC-3′

### 2.9 Western blot

Protein with equal concentrations in each group were mixed with 5× protein loading buffer (Sigma-Aldrich, United States) and incubated at 99°C for 10 min. Then running the sample by 15% SDS-PAGE gels before being subsequently transferred to NC membranes (GE, United States). The immnunoblots were incubated with 5% milk at room temperature for 0.5–1 h, followed by an incubation at 4°C with primary antibodies overnight. After rinsing the membranes five times with TNET buffer (5 min of each), incubated with IgG (H + L)-HRP secondary antibodies (Santa Cruz, United States) for 1–2 h. The band densities were detected using an LumiBest ECL solution from Shanghai Sharebio Biotechnology (Shanghai, China) and Bio-Rad Laboratories (BIO-RAD, United States). ImageJ Software was using for analysis of the results.

### 2.10 Myeloperoxidase activity

For the purpose of measuring myeloperoxidase (MPO) activity, the lung tissues of mice were weighted and homogenized. Referring to the Myeloperoxidase (MPO) assay kit from Nanjing Jiancheng (Nanjing, China), the activity of MPO was measured.

### 2.11 Determination of NO level

BMDMs were plated and pre-treated with LMZ 0, 3, 10 or 30 μM for 30 min and challenged with 100 ng/mL LPS for 24 h to collect the supernatant. Nitric oxide (NO) levels in BMDMs were tested by NO Kit (Beyotime, Shanghai, China).

### 2.12 Ca^2+^ influx imaging

The pre-cultured BMDMs were washed three times with HBSS. The Fluo-4-AM 4 μM working solution (Shanghai Sharebio Biotechnology, China) was added to the cells and incubated at 37°C for 40 min. Then removed Fluo-4-AM green-fluorescent calcium indicator and rewashed the cells with HBSS. The cells were then incubated with or without LMZ (30 μM) at 37°C for 10 min before exposed to LPS (1 μg/mL) and Ca^2+^ channel agonist Bay K8644 (BK, 10 μM). Fluorescent Ca^2+^ was detected under a laser-scanning confocal fluorescence microscope (Zeiss LSM 900, Germany). Fluo-4-AM was performed for 10 min (6s intervals) via fluorescence excited at 494 nm and collect fluorescence emission at 516 nm for calcium imaging.

### 2.13 Statistical analysis

Data shown mean ± standard deviation (s.d.) of one representative experiment out of three independent *in vivo* experiments and shown mean ± standard error of the mean (s.e.m) of three independent *in vitro* experiments. All Data were analyzed using one-way ANOVA (Tukey’s test) with Prism version 9 (GraphPad Software, San Diego, CA, United States). *p*-values <0.05 were considered to be statistically significant.

## 3 Result

### 3.1 Lomerizine attenuates LPS-induced acute lung injury

**FIGURE 1:**
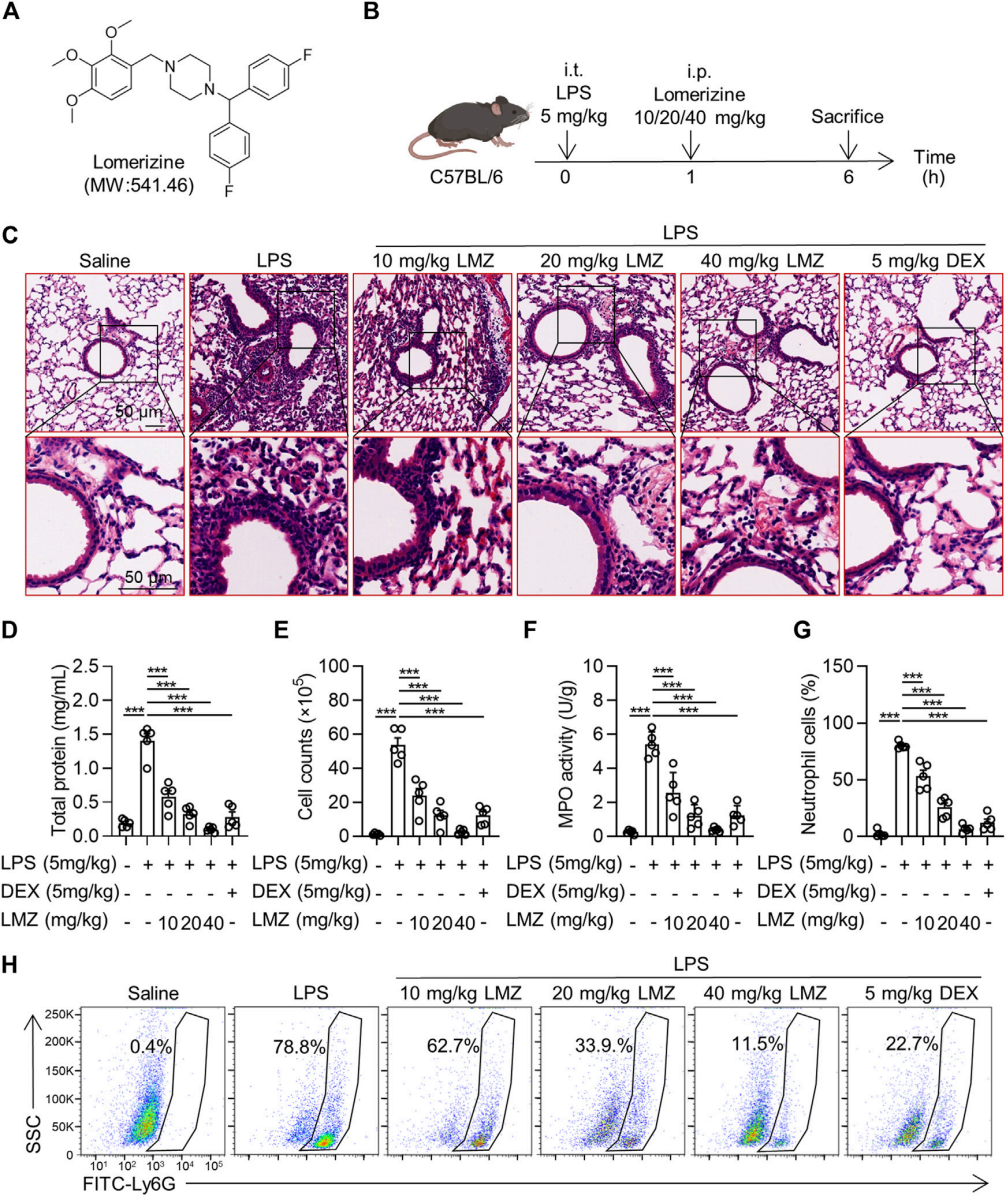
Lomerizine (LMZ) attenuated lipopolysaccharide (LPS)-induced acute lung injury (ALI). **(A)** Chemical structure of LMZ. **(B)** Experimental schematic of LPS-induced mice receiving treatment of LMZ. 30 mice were randomly assigned into PBS group, LPS group (5 mg/kg), LMZ group (10, 20 and 40 mg/kg) and dexamethasone group (DEX, 5 mg/kg). After anesthesia, mice were intraperitoneally injected with different concentrations of LMZ and DEX 1 h after intratracheal injection of LPS and subsequent experiments were performed 5 h later. **(C)** Representative images of lung sections were taken by orthomosaic microscopic observation. LPS-induced bronchoalveolar lavage fluid (BALF) in mice was detected to validate the ALI model. **(D)** Total protein levels in BALF. **(E)** Total cell counts in BALF. **(F)** Myeloperoxidase (MPO) activity in the lungs. **(G)** Statistical analysis of Figure 1H. **(H)** Levels of the neutrophil marker Gr-1 in BALF were examined by flow cytometry. Data shown mean ± s.d. of one representative experiment out of three independent experiments. n = 5 per group, *p*-values were calculated by one-way ANOVA (Tukey’s test). ***p < 0.001.

To determine the function of lomerizine (LMZ) on acute lung injury (ALI), We established lipopolysaccharide (LPS)-induced ALI mice and treated them with LMZ and dexamethasone (DEX) (Figure 1B). Hematoxylin and eosin (HE) staining of lung tissue showed alveolar wall thickening, lung tissue destruction and infiltration of inflammation cells deteriorated in the LPS-treated lung tissue (Figure 1C). However, LMZ treatment significantly reduced the infiltration of inflammatory cells and protected alveolar structures in LPS-induced ALI mice. The protein concentration in bronchoalveolar lavage fluid (BALF) of ALI mice was increased significantly compared with that of the PBS group (Figure 1D). Meanwhile, the number of cells in BALF of LPS-induced mice was increased significantly (Figure 1E). Myeloperoxidase (MPO) activity was measured as a marker of neutrophil infiltration. LPS-challenged mice showed a significant increase in MPO activity in lung tissue, while LMZ treatment significantly inhibited MPO activity (Figure 1F). We detected Ly6G-positive number to assess the infiltration of neutrophils in lung tissue (Figure 1G). LMZ treatment significantly decreased Ly6G-positive neutrophils in BALF (Figure 1H). These data indicated that LMZ mitigates LPS-induced ALI.

### 3.2 Lomerizine inhibits macrophage activation in LPS-induced mice through NK-κB and MAPK signal pathways

**FIGURE 2:**
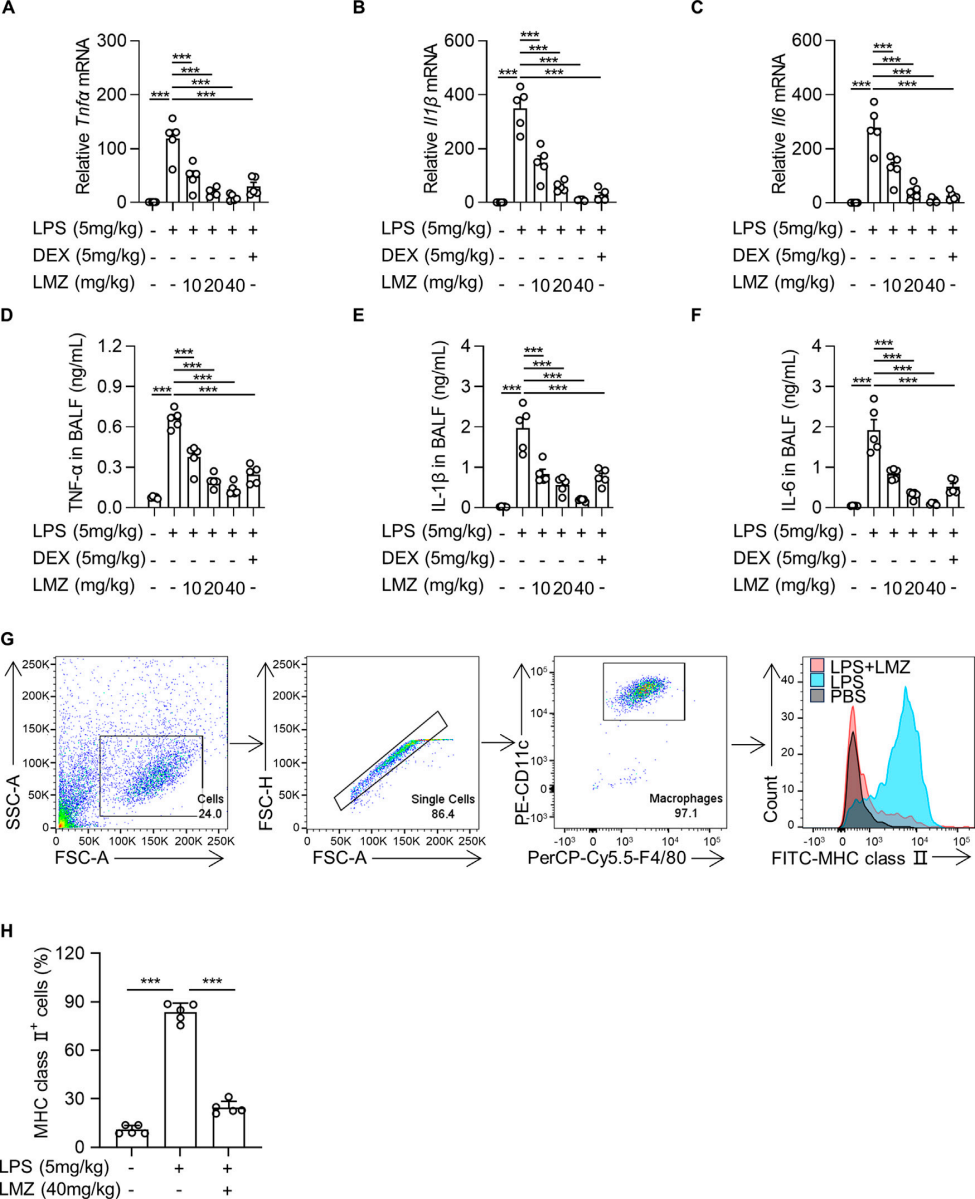
Lomerizine (LMZ) reduced the production and secretion of pro-inflammatory cytokines in lipopolysaccharide (LPS)-induced acute lung injury mice. LMZ (0, 10, 20 and 40 mg/kg) or DEX (5 mg/kg) treatments were administered 1 h after LPS stimulation (5 mg/kg). **(A–C)** The mRNA levels of *Tnfα, Il1β* and *Il6* were examined in mice lung tissue by real-time polymerase chain reaction (RT-PCR). **(D–F)** BALF was collected and ELISA kits detected the protein expression level of each pro-inflammatory cytokines. **(G)** The percentage of alveolar macrophages in BALF after LMZ (40 mg/kg) treatment were examined by flow cytometry. **(H)** The percentage of MHC class Ⅱ-positive alveolar macrophages in BALF. Data shown mean ± s.d. of one representative experiment out of three independent experiments. n = 5 per group, *p*-values were calculated by one-way ANOVA (Tukey’s test). ***p < 0.001.

To further investigate the phenotype of LMZ in alleviating LPS-induced ALI, we measured the expression levels of pro-inflammatory cytokines of LPS-induced mice in BALF by RT-PCR. The mRNA expression levels of TNF-α, IL-1β and IL-6 were significantly upregulated by LPS treatment, while suppressed by LMZ treatment dose-dependently, which was similar with dexamethasone (DEX) (Figures 2A–C). Consistently, the enzyme-linked immunosorbent assay (ELISA) results showed that LMZ dose-dependently reduced the protein levels of TNF-α, IL-1β and IL-6 induced by LPS (Figures 2D–F). Macrophages play a critical role in the pulmonary inflammation and are involved in tissue injury and inflammatory recovery processes (Chen et al., 2001). To determine whether LMZ alleviates macrophage activation, we stained the cells from BALF with PE-CD11c, PerCP-Cy5.5-F4/80 and FITC-MHC class II antibodies. Alveolar macrophages can be considered as CD11c^+^ F4/80^+^ cells. MHC class II reflected activation of macrophage after LPS stimulation. Flow cytometry analysis revealed an increased percentage of CD11c^+^ F4/80^+^ MHC class Ⅱ^+^ cells after LPS stimulation in BALF which was restored after LMZ treatment (Figure 2G). Moreover, compared to PBS group, the percentage of MHC class Ⅱ-positive cells were approximately 9-fold increase in LPS group, but only about 2-fold increase in LPS and LMZ co-administration group (Figure 2H). These results indicated that LMZ inhibits macrophage activation in LPS-induced mice.

**FIGURE 3:**
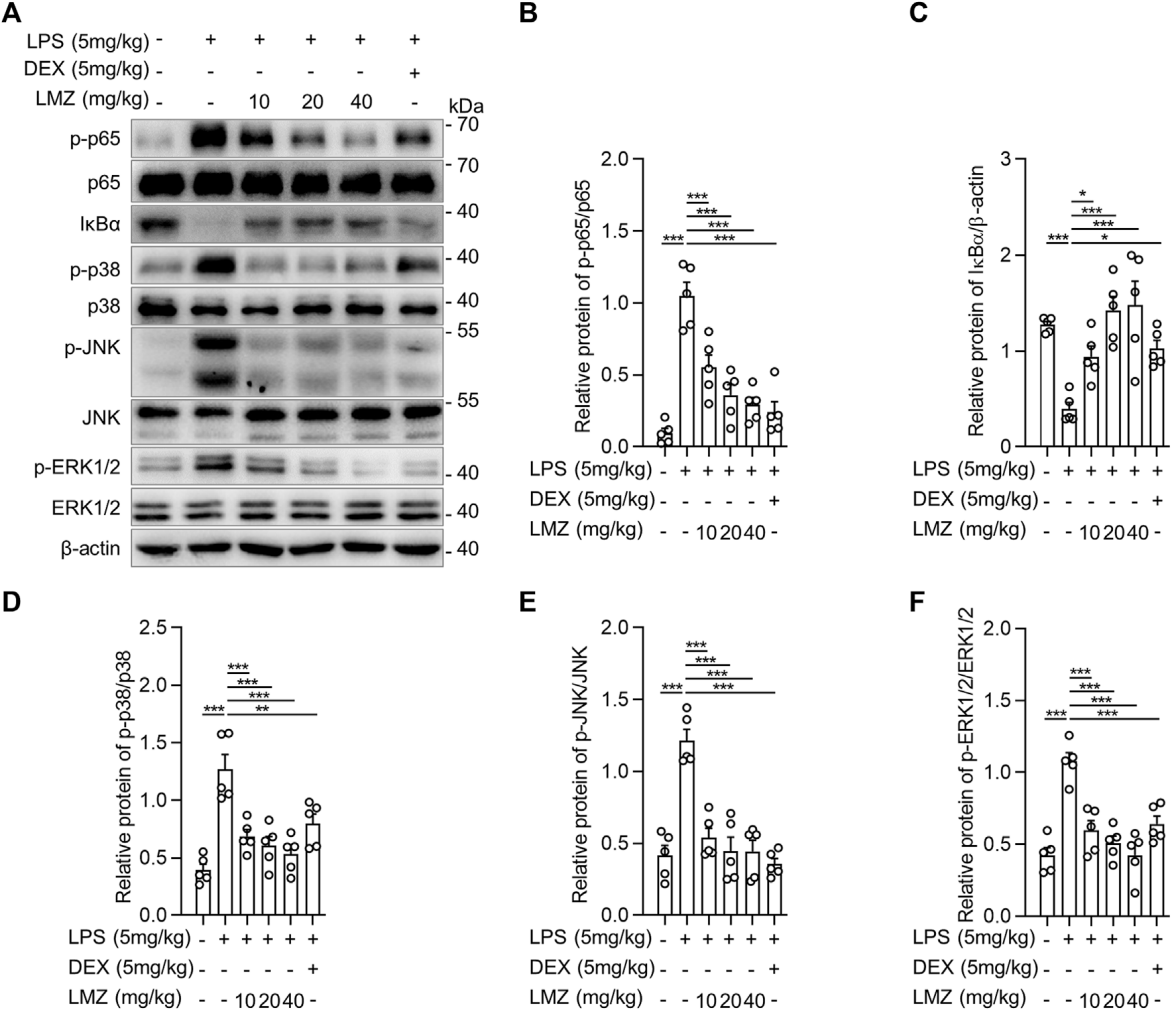
Lomerizine (LMZ) suppressed inflammation through NK-κB and MAPK signal pathways in lipopolysaccharide (LPS)-induced acute lung injury mice. **(A)** The phosphorylation level of NF-κB and MAPK kinases by Western blotting. The ratios of the grayscale values of the bands, **(B)** phosphor-p65 (p-p65) and total p65, **(C)** IκBα and β-actin, **(D)** p-p38 and total p38, **(E)** p-JNK and total JNK and **(F)** p-ERK1/2 and total ERK1/2. Data shown mean ± s.d. of one representative experiment out of three independent experiments. n = 5 per group, *p*-values were calculated by one-way ANOVA (Tukey’s test). *p < 0.05, **p < 0.01, ***p < 0.001.

Toll-like receptor 4 (TLR4), which is a classical pattern recognition receptor, plays a key role in the activation of innate immunity (Vijay, 2018; Sartorius et al., 2021). Mitogen-activated protein kinase (MAPK) and nuclear factor kappa B (NF-κB) are two crucial downstream signaling pathways, which contribute to TLR4-induced pro-inflammatory cytokine production (Kawasaki and Kawai, 2014; Manik and Singh, 2022). To determine the underlying anti-inflammatory mechanism of LMZ, we measured the expression and phosphorylation levels of p65 in NF-κB signal pathway, ERK1/2, p38 MAPK, and JNK in MAPK signal pathway in lung tissues of LPS-induced mice. The phosphorylation level of p65 in lung tissue was remarkably increased after LPS stimulation and gradually decreased after LMZ treatment (Figures 3A,B). To further confirm the negative regulator of LMZ in NF-κB pathway, we investigated the expression levels of NF-κB specific inhibitor IκBα, a classical inhibitor of NF-κB. As shown in Figure 3C, the expression level of IκBα was decreased after LPS stimulation, while enhanced by co-administration with LMZ dose-dependently. Furthermore, the phosphorylated level of p38 MAPK, ERK1/2, and JNK increased significantly after LMZ treatment dose-dependently (Figures 3D–F). These results suggested that LMZ significantly attenuated the production of inflammatory cytokines through MAPK and NF-κB signal pathways in LPS-induced ALI mice.

### 3.3 Lomerizine inhibits the pro-inflammatory cytokine production through MAPK and NF-κB signal pathways in macrophages

**FIGURE 4:**
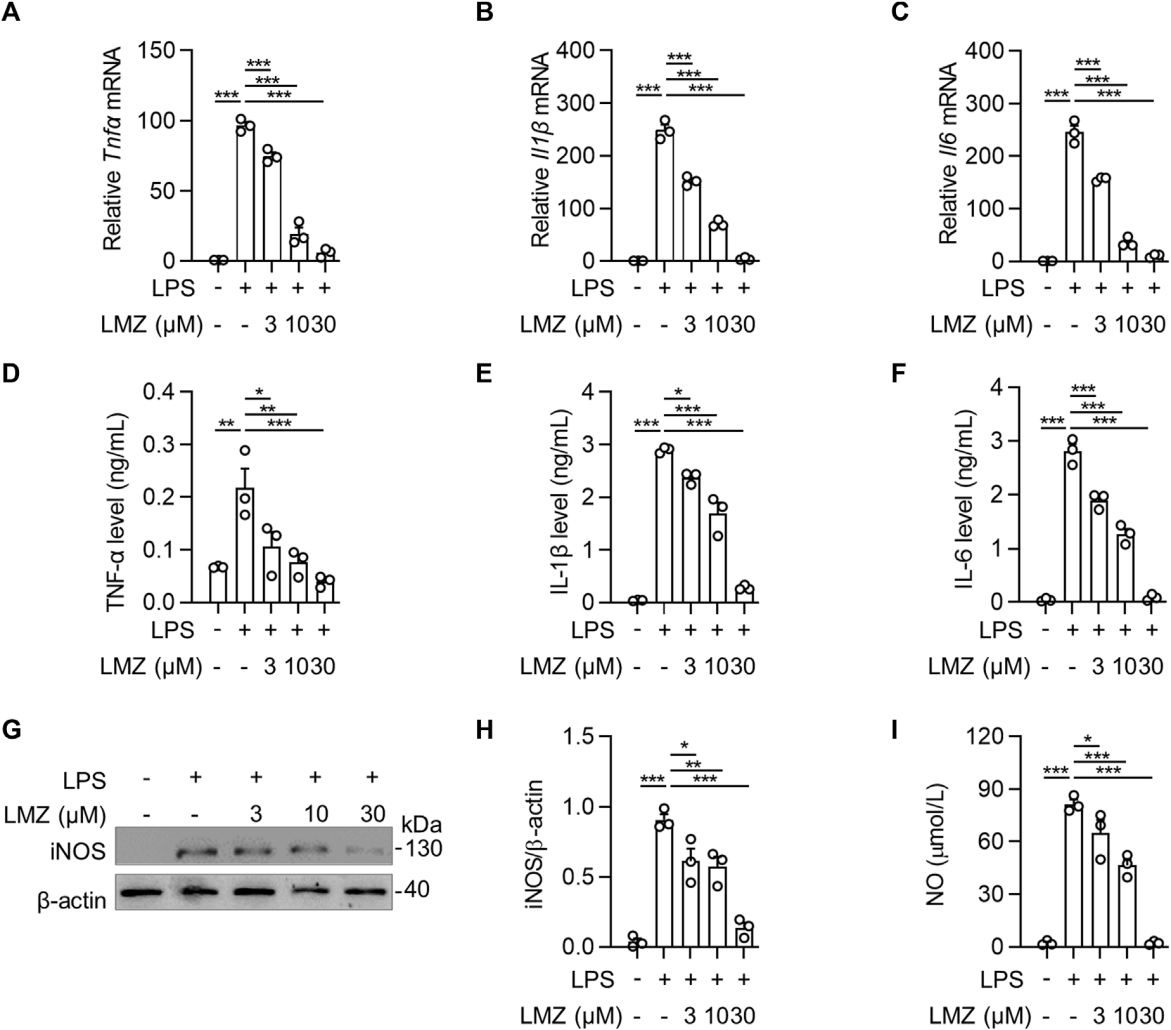
Lomerizine (LMZ) inhibited the production of pro-inflammatory cytokines in macrophages. BMDMs were pretreated with LMZ (0, 3, 10 and 30 μM) for 30 min before 4 h LPS challenge (100 ng/mL). The mRNA expression levels of *Tnfα* **(A)**, *Il1β* **(B)** and *Il6* **(C)** in BMDM cell lysates were detected by RT-PCR. All groups were normalized to PBS group. The protein levels of TNF-α **(D)**, IL-1β **(E)** and IL-6 **(F)** in the supernatant of the culture medium were measured by ELISA kits. **(G–H)** The protein levels of iNOS in BMDMs were tested by Western blotting. **(I)** NO levels in BMDMs were tested by NO assay kit. Data shown mean ± s. e.m of three independent experiments. *p*-values were calculated by one-way ANOVA (Tukey’s test). *p < 0.05, **p < 0.01, ***p < 0.001.

**FIGURE 5:**
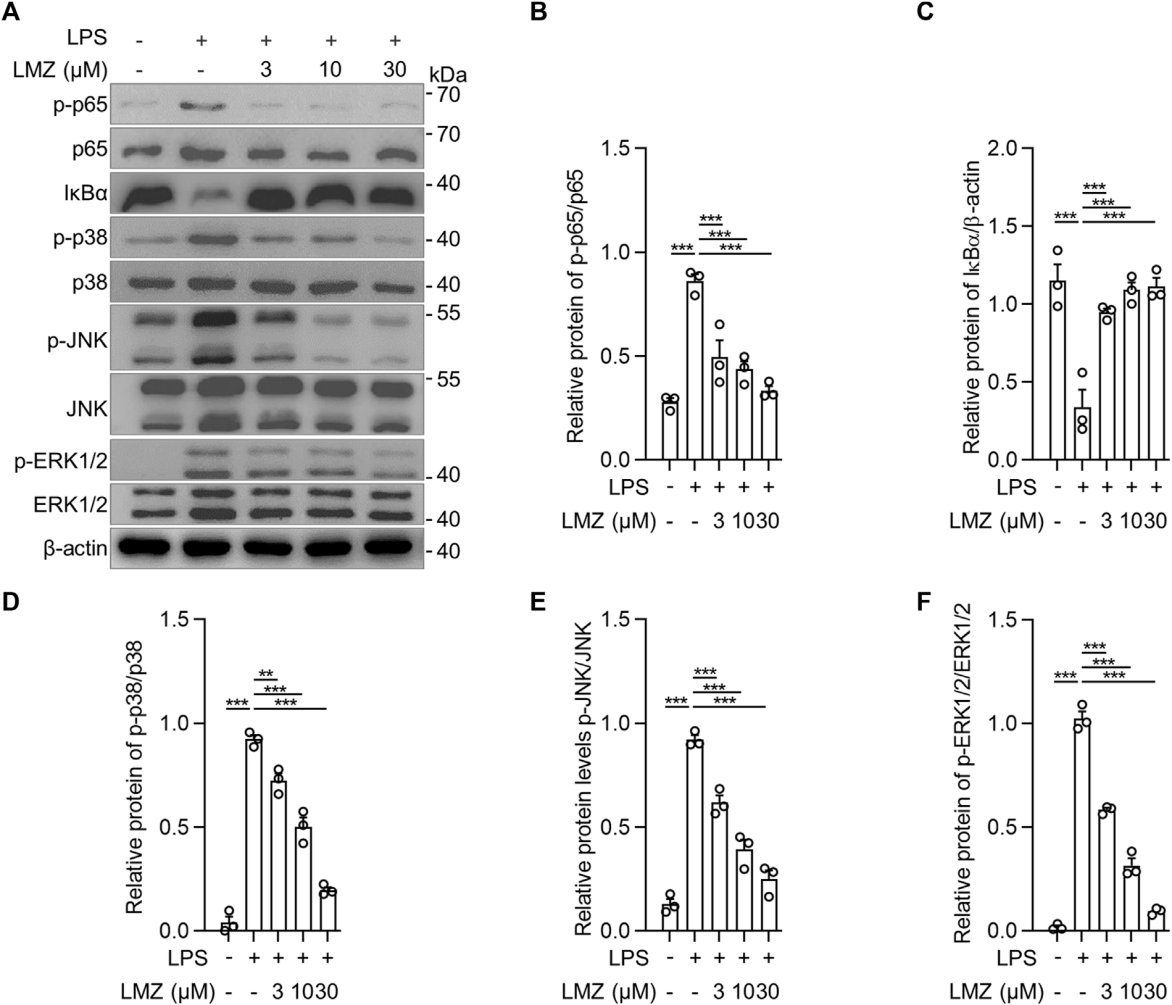
Lomerizine (LMZ) decreased the phosphorylation levels of NF-κB and MAPK pathways in macrophages. BMDMs were pretreated with LMZ (0, 3, 10 and 30 μM) for 30 min before 4 h LPS challenge (100 ng/mL). **(A)** The phosphorylation levels of NF-κB and MAPK kinases in BMDM cell lysates by Western blotting. **(B)** p-p65 and total p65, **(C)** Iκbα and β-actin, **(D)** p-p38 and total p38, **(E)** p-JNK and total JNK, **(F)** p-ERK1/2 and total ERK1/2. The ratio of grayscale values of the bands was quantified using ImageJ software. Data shown mean ± s. e.m of three independent experiments. *p*-values were calculated by one-way ANOVA (Tukey’s test). ***p < 0.001.

To determine the role of inflammatory cytokines induced by LPS in macrophages, we detected the expression of inflammatory cytokines using RT-PCR and ELISA in BMDMs. The results showed that the mRNA levels of pro-inflammatory cytokines were dramatically upregulated in LPS-stimulated BMDMs compared to that of the PBS group, while the expression levels were downregulated with LMZ treatment dose-dependently (Figures 4A–C). Similarly, the protein levels of TNF-α, IL-1β and IL-6 in macrophages increased after LPS challenge (Figures 4D–F). Furthermore, we also measured downstream mediators of inflammatory cytokines, inducible nitric oxide synthase (iNOS) and NO, expression levels in BMDMs. The expression levels of iNOS and the concentration of NO were significantly upregulated in BMDMs after LPS stimulation (Figures 4G–I). Compared with the LPS group, pre-treated with LMZ downregulated the expression of iNOS and NO dose-dependently. Furthermore, we measured the activity of NF-κB and MAPK signal pathways in LPS-induced macrophages pretreated with LMZ. Results showed that LMZ significantly inhibited the phosphorylated level of p65 and increased the protein level of IκBα (Figures 5A–C). Similarly, the phosphorylated level of p38 MAPK, JNK, ERK1/2 in MAPK signal pathway were significantly decreased after LMZ pretreatment in LPS-challenged BMDMs (Figures 5D–F). These results show that LMZ alleviated LPS-induced pro-inflammatory response through MAPK and NF-κB pathways.

### 3.4 Lomerizine reduces the expression of pro-inflammatory cytokines via the Ca^2+^ pathway in macrophages

**FIGURE 6:**
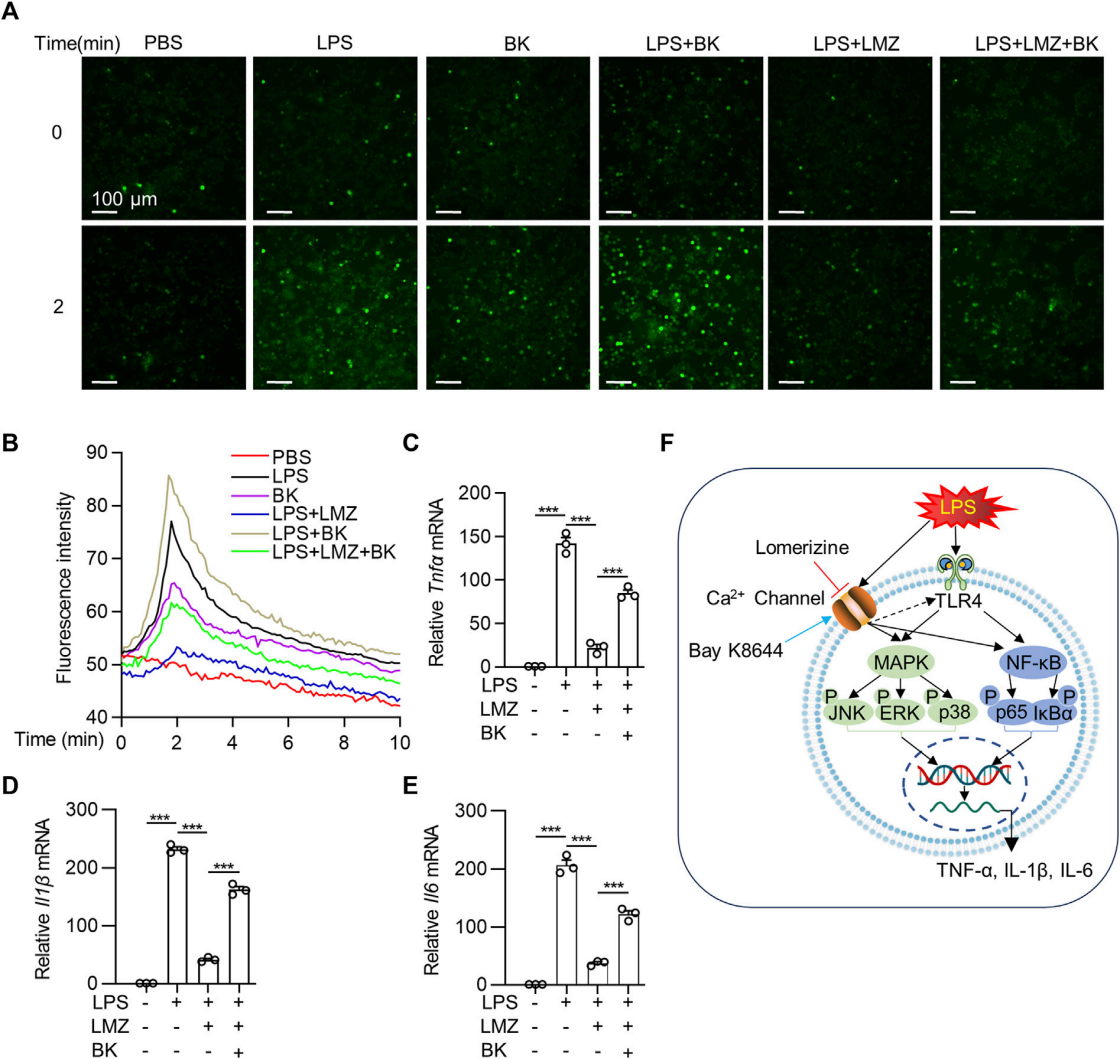
Lomerizine (LMZ) reduced the expression of pro-inflammatory cytokines via the Ca^2+^ pathway in macrophages. BMDMs were pretreated with Fluo-4 AM ester solution for 30 min. Cells were then treated with LMZ alone or LMZ co-treated with Bay K8644 (BK, 10 μM) before exposed to lipopolysaccharide (LPS, 100 ng/mL). **(A)** Ca^2+^ was detected as fluorescent bright green using laser scanning confocal fluorescence microscopy in 10 min in BMDMs. **(B)** Green fluorescence intensity was quantified. Scale bar, 100 μm. BMDMs were pretreated with LMZ (10 μM) alone or co-treated BK (10 μM) and LMZ (10 μM) followed by LPS stimulation for 4 h. The mRNA levels of *Tnfα* **(C)**, *Il1β* **(D)** and *Il6* **(E)** were detected in BMDMs by RT-PCR. **(F)** Schematic illustration of possible mechanisms by which lomerizine (LMZ) inhibited acute lung injury mediated NF-κB and MAPK signaling in macrophages. Data shown mean ± s. e.m of three independent experiments. *p*-values were calculated by one-way ANOVA (Tukey’s test). ***p < 0.001.

LPS has been reported to promote Ca^2+^ influx in macrophages (Chen et al., 2001; Letari et al., 1991; Zhou et al., 2006). To determine the association between Ca^2+^ influx and pro-inflammatory cytokines expression in macrophages, we detected Ca^2+^ influx using a Fluo-4 AM green-fluorescent calcium indicator. Compared with PBS group, fluorescence intensity increased sharply within 2 min after LPS or Ca^2+^ channel agonist Bay K8644 (BK) stimulation, which was decreased after LMZ pretreatment in BMDMs (Figures 6A,B). Interestingly, we co-incubated LMZ with BK in LPS-induced BMDMs, the fluorescence intensity increased within 2 min again. Moreover, the expression of *Tnfα*, *Il1β*, and *Il6* were successfully reduced by LMZ and reversed by BK treatment in BMDMs (Figures 6C–E). Namely, LMZ attenuated LPS-induced inflammation by inhibiting Ca^2+^ influx in BMDMs (Figure 6F). These data suggested that the anti-inflammatory effect of LMZ rely on Ca^2+^ channels activation.

## 4 Discussion

Acute lung injury (ALI)/acute respiratory distress syndrome (ARDS) is an extremely hazardous pulmonary inflammatory syndrome. ALI is a respiratory system disease with respiratory distress with a PaO_2_/FiO_2_ ≤ 300 mmHg in clinical defined by American-European Consensus Committee (AECC) in 1994 (Bernard et al., 1994). The majority of acute lung injury models are constructed on the basis of their pathological characteristics, which include pulmonary neutrophil infiltration, hyaline membrane deposition, and microthrombus formation (Matute-Bello et al., 2008). LPS challenge is widely used to mimic human ALI. Upon intratracheal instillation with LPS in mice, the expression of proinflammatory cytokines reach the peak around 6 h and then gradually declined, which could be used to determine the potential reagents to alleviate ALI (Li et al., 2012; Jing et al., 2015; Wu et al., 2018). Corticosteroids, an effective anti-inflammatory medicine, have been wildly used in treating ALI/ARDS. However, corticosteroids treatment is always associated with neuromuscular complications and may cause myoglobulin loss in severely ill patients (Amaya-Villar et al., 2005; Rady et al., 2006; Liu et al., 2013). In this study, we found that lomerizine (LMZ) significantly alleviated lipopolysaccharide (LPS)-induced ALI both *in vitro* and *in vivo*, which showed LMZ is a potential therapeutic drug for ALI/ARDS treatment.

Ca^2+^ influx is one of the most important cell signals for regulating various physiological and pathological processes, such as cancer, heart failure, diabetes and neurodegenerative disease (Rossi et al., 2019; Varghese et al., 2019). In recent years, accumulating literature have highlighted the link between Ca^2+^ influx and inflammation. Saddala et al. found that blocking Ca^2+^ influx inhibited the activation of calcineurin (CN) and recruitment of IκB kinases (IKK) through L-type voltage-gated calcium channel (L-VGCC), resulting in the inhibition of nuclear factor kappa B (NF-κB) activation (Saddala et al., 2020). Tauseef revealed that activated Ca^2+^ influx into endothelial cells (EC) via myeloid differentiation factor 88 (MyD88) and NF-κB pathway eventually inducing inflammation (Tauseef et al., 2012). Here, we validated the inflammatory therapeutic effect of LMZ on ALI by using LPS-induced ALI mice. Furthermore, In LPS-stimulated BMDMs, LMZ treatment significantly inhibited pro-inflammatory cytokines expression. While the Bay K8644 (BK) treatment significantly increased inflammatory cytokines expression, which were suppressed by LMZ. These results indicated that LMZ inhibited the pro-inflammatory process via Ca^2+^ influx, providing evidence for studying the relationship between Ca^2+^ influx and inflammation.

Activation of mitogen-activated protein kinase (MAPK) and NF-κB signaling pathways increase the expression of cytokines in pneumonia and aggravate ALI. The activation of MAPK can promote the induction of inflammatory factors, COX-2 and iNOS, thus up-regulating the inflammatory reactivity (Choi et al., 2009; Bhatt et al., 2010). Several studies have shown that LPS binding to TLR4 activates downstream NF-κB and MAPK signaling pathways in macrophages (Lu et al., 2008; Li et al., 2015). IκBα is an essential negative signaling factor in NF-κB signaling pathway, which segregates NF-κB and p65 complex (Solt and May 2008; Oeckinghaus and Ghosh, 2009). LMZ decreased the phosphorylation level of p65 in NF-κB and p38 MAPK, JNK, and ERK1/2 in MAPK pathway and increased the expression level of IκBα in BMDMs. Our results demonstrated that LMZ ameliorated ALI by inhibiting inflammatory signaling through the MAPK and NF-κB pathways in macrophages which could guide future studies in this area.

Previous studies have found therapeutic effects of lomerizine (LMZ) in cardiovascular diseases. Shimazawa et al. found that LMZ inhibited hypoperfusion and expression of c-Fos-like immunoreactivity on the cortical to treat migraine (Shimazawa et al., 1995). Concerning the protective effects of lomerizine on neuronal, Toriu et al. found that LMZ protected neuronal cells by inhibiting glutamate-induced neurotoxicity, ischemia, and reperfusion damage (Toriu et al., 2000). Moreover, Fitzgerald et al. validated the therapeutic effect of LMZ in optic nerve (ON) injury rats by inhibiting L-type calcium channels on the RGC (Fitzgerald et al., 2009). The role of LMZ in apoptosis is intriguing, but we did not find LMZ to induce apoptosis in our dose range, which is consistent with existing studies (Ito et al., 2010; Park et al., 2023). In this study, we found that LMZ alleviates acute lung injury by decreasing expression of inflammatory factors in macrophages which broadens the potential applications of LMZ in the field of pulmonary inflammatory diseases.

In conclusion, our data reveal an injury-preventive role of LMZ in the LPS-induced mice model. We found that LMZ inhibited the phosphorylation level of the NF-κB and MAPK pathway by blocking Ca^2+^ influx in macrophages, thereby reducing the release of pro-inflammatory factors, indicating that it is a potential agent in treating acute lung injury.

## Data Availability

The original contributions presented in the study are included in the article/Supplementary Material, further inquiries can be directed to the corresponding authors.
